# Integrated Transcriptomic Analysis Identifies Novel Candidate Genes Associated with Calcific Aortic Valve Disease

**DOI:** 10.3390/genes17020246

**Published:** 2026-02-20

**Authors:** Jing Chen, Shichao Guo, Junming Zhu, Haiou Hu, Bing Tang, Lingchen Huang, Chenhan Zhang, Suwei Chen, Sanbao Chai, Zhiyu Qiao, Hongfeng Jiang

**Affiliations:** 1 Beijing Anzhen Hospital of Capital Medical University and Beijing Institute of Heart Lung and Blood Vessel Diseases, Beijing 101118, China; 2 Center for Aortic Surgery, Beijing Anzhen Hospital of Capital Medical University, Beijing 101118, China; 3 Department of Endocrinology and Metabolism, Peking University International Hospital, Beijing 102206, China

**Keywords:** calcified aortic valve disease (CAVD), machine learning, single-cell RNA sequencing, osteogenesis, BAMBI, HAND2, MYOC

## Abstract

Background: Calcified aortic valve disease (CAVD) is a prevalent valvular disorder in the elderly and a major cause of aortic stenosis. Surgical and transcatheter aortic valve replacement remain the primary treatments for advanced CAVD; however, effective pharmacological therapies to prevent or slow disease progression are lacking. Therefore, there is an urgent need to explore potential novel candidate biomarkers and therapeutic targets. Methods: In this study, transcriptomic data from multiple independent datasets were integrated to comprehensively characterize the transcriptional profile of CAVD. Feature genes were identified using complementary machine learning approaches, followed by functional pathway enrichment and protein–protein interaction (PPI) network analyses to uncover novel candidate genes associated with CAVD. Single-cell RNA sequencing (sc-RNA-Seq) data were further analyzed using pseudotime trajectory analysis to explore transcriptional dynamics during valve interstitial cells’ (VICs) osteogenic progression. Quantitative PCR and Western blot analyses of human calcified aortic valve tissues were used for validation. Results: A total of 119 CAVD-associated genes were identified, primarily involved in ossification, extracellular matrix organization, and cell–substrate adhesion. Among these, the ossification-associated genes BAMBI, HAND2, and MYOC exhibited potential discriminatory power between CAVD and control samples, with notable downregulation in calcified valves. Pseudotime analysis showed that the expression of these genes gradually decreased along the transcriptional trajectory associated with osteogenic differentiation. In addition, the analysis of relative immune signatures revealed negative correlations between these genes and multiple immune signatures. Conclusions: This study identifies novel candidate genes underlying CAVD pathogenesis and highlights BAMBI, HAND2, and MYOC as potential biomarkers and therapeutic targets, providing new insights into disease mechanisms and opportunities for novel interventions.

## 1. Introduction

Calcified aortic valve disease (CAVD) is a common cardiovascular disease with high morbidity and mortality [1,2,3]. It is characterized by thickening and calcification of the aortic valve, which further progresses into a series of atypical hemodynamic changes and, eventually, severe aortic valve stenosis [4]. Currently, there are no effective pharmacological interventions to terminate the progression of CAVD [5]. Surgical and transcatheter aortic valve replacement (TAVR) remain the standard treatment for severe aortic stenosis patients, which often causes complications and provides poor long-term outcomes [4,6,7,8]. Consequently, this unaddressed clinical exigency has instigated further comprehensive investigations into the mechanisms underlying CAVD, aiming to formulate novel therapeutic approaches.

CAVD was historically regarded as a passive degenerative disorder caused by mechanical wear and tear. However, growing evidence now indicates that it represents an active and highly regulated biological process involving inflammation, immune cell infiltration, lipid accumulation, and the osteogenic differentiation of valvular interstitial cells (VICs). The gross pathology of valve calcification shares striking similarities with atherosclerosis. One of the earliest events is endothelial disruption on the aortic side of the valve leaflets [9], followed by progressive matrix remodeling driven by metalloproteinases and age-related structural alterations. These changes result in leaflet thickening, referred to as aortic valve sclerosis, which is typically asymptomatic. As the disease advances, immune cell infiltration, angiogenesis, and the deposition of lipids, proteoglycans, and cellular debris contribute to leaflet deformation. Eventually, the valve matrix undergoes calcification, leading to aortic stenosis and the obstruction of blood flow [10,11].

At the cellular level, VICs undergo myofibroblastic and osteogenic phenotypic transitions during disease initiation and progression, driving extracellular matrix (ECM) remodeling, collagen deposition, and the formation of bone-like tissue within the valve [1]. Notably, approximately 13% of calcified aortic valves obtained at the time of surgery have been found to contain true bone tissue, complete with osteoblasts and osteoclasts, underscoring the pathological relevance of osteogenesis in CAVD [9]. Despite these advances in understanding the pathological landscape of the disease, the key genes and molecular mechanisms that regulate VIC osteogenic differentiation remain poorly characterized.

Therefore, in the present study, we integrated public human CAVD-related bulk RNA sequencing (RNA-Seq) and single-cell RNA sequencing (scRNA-Seq) datasets and conducted a comprehensive series of bioinformatics analyses to identify novel candidate genes associated with osteogenic processes in CAVD. Through this integrative approach, we aimed to identify genes associated with valve calcification, serving as a basis for further exploration of promising biomarkers and potential therapeutic targets.

## 2. Methods

### 2.1. Data Curation

Transcriptomic data analyzed in this study were obtained from three publicly available repositories: the CICS MultiOmics Databases (Brigham and Women’s Hospital, Harvard Medical School), the Gene Expression Omnibus (GEO; National Center for Biotechnology Information, USA), and the EMBL-EBI Expression Atlas. These databases were systematically searched to identify RNA sequencing datasets related to calcific aortic valve disease CAVD.

Datasets were included if they met the following criteria: (1) samples were derived from human aortic valve tissue; (2) each dataset contained both calcified aortic valve samples and non-calcified (healthy) controls; and (3) each dataset included at least four samples in total. Based on these criteria, seven eligible bulk RNA-Seq datasets were included in this study: GSE148219, GSE153555, GSE199718, GSE235995, GSE55492, and E-MTAB-11354, as well as one dataset of CAVD layers obtained from the CICS MultiOmics database (Supplementary Table S1) [12,13,14,15,16,17,18]. For convenience, this CICS dataset is referred to as “CICSharvard” in this study.

### 2.2. Preprocessing of RNA Sequencing Datasets

Raw RNA-Seq count matrices were obtained from seven publicly available datasets, as described in the data curation subsection. Based on the clinical information, RNA-Seq samples were assigned into a control group and a CAVD group.

Batch effects across datasets were corrected using the ComBat-Seq method, which adjusts for technical variability while preserving the count-based structure of RNA-Seq data. Principal component analysis (PCA) was performed before and after batch correction to visually assess the effectiveness of batch effect removal. The batch-corrected count data were subsequently used for downstream differential expression analysis between the control and CAVD groups. These corrected counts were also transformed into transcripts per million (TPM) values for the relative immune signatures analysis.

In addition, the uncorrected raw count data were randomly split into a training set and a testing set at a ratio of 6:4 with balanced class distribution. Batch correction was applied separately to each subset using ComBat-Seq to avoid information leakage. Ma-chine learning models were trained on the batch-corrected training set to identify feature genes, and model performance was evaluated on the independently batch-corrected testing set using receiver operating characteristic (ROC) curve analysis.

### 2.3. Differential Expression Analysis

Differential expression analysis was conducted using the DESeq2 package (v1.44.0) in R (v4.5.0). Batch-corrected count matrices from all RNA-Seq samples were imported for analysis. Genes with a Benjamini–Hochberg (BH) false discovery rate (FDR)-corrected *p*-value < 0.05 and an absolute log_2_ fold change (|log_2_FC|) > 1 were considered significantly differentially expressed.

### 2.4. Functional Enrichment Analysis

Gene Ontology (GO) functional enrichment analysis and Kyoto Encyclopedia of Genes and Genomes (KEGG) pathway analysis were performed using the “enrichGO” and “enrichKEGG” functions of the clusterProfiler (v4.12.6) R package respectively. GO terms and KEGG pathways with a BH FDR-corrected *p*-value < 0.05 were considered significantly enriched.

### 2.5. Machine Learning

Batch-corrected RNA-Seq count data from the training set were used to identify candidate genes associated with CAVD using three complementary machine learning approaches.

A support vector machine with a linear kernel (SVM-Linear) was implemented using the e1071 (v1.7.16) and caret (v7.0.1) R packages. Recursive feature elimination (RFE) was applied to rank genes based on their contribution to classification performance. Candidate subsets of predefined sizes (50, 100, 150, and 200 genes) were evaluated to determine the optimal number of genes that maximized classification accuracy. Model training and feature selection were conducted using fivefold cross-validation with three repeated runs to improve robustness and reduce overfitting. The cost parameter, which controls the trade-off between margin maximization and classification error, was tuned within the cross-validation framework. Genes retained in the optimal feature subset were considered candidate genes identified by the SVM-Linear approach.

Random forest (RF) analysis was conducted using the caret R package. The model was trained using fivefold cross-validation with three repeated runs, and class probabilities were estimated to improve model calibration and performance assessment. Feature importance was calculated during model training, and genes were ranked based on their contribution to classification accuracy. Fifty genes with the highest importance scores were selected as candidate genes identified by the RF model.

Extreme gradient boosting (XGBoost) was performed using the xgboost (v1.7.11.1) R package. The gradient-boosted decision tree model was trained using fivefold cross-validation with three repeated runs. A maximum tree depth of three to limit model complexity and reduce overfitting, a learning rate of 0.1 to control the contribution of each boosting iteration, and 100 boosting rounds were adopted to ensure adequate model convergence. A binary logistic objective function was used for classification. Feature importance was assessed using the gain metric, representing the relative contribution of each gene to improving model performance, and 50 genes with the highest gain values were identified as candidate genes by the XGBoost model.

GO pathway enrichment analysis was performed on the union of candidate genes of the three models, with the top-ranked ossification pathway identified. Eleven genes associated with this pathway were selected for further investigation.

### 2.6. Odds Ratio Calculation for Candidate Genes

Logistic regression analysis was performed to evaluate the association between gene expression levels and disease status. For each candidate gene, a generalized linear model (GLM) with a binomial distribution and logit link function was fitted, using the log_2_-transformed batch-corrected counts (log_2_(count + 1)) as the predictor and disease status (CAVD vs. control) as the outcome. Regression coefficients and standard errors were extracted from each model. Odds ratios (ORs) were calculated by exponentiating the regression coefficients. The 95% confidence intervals (CIs) were derived as OR ± 1.96 × standard error on the log scale and then exponentiated. Statistical significance was assessed using the Wald test.

### 2.7. Protein–Protein Interaction

Genes associated with the ossification pathway were used to construct a protein–protein interaction (PPI) network in R based on known and predicted interactions taken from the STRING database (confidence score > 0.4).

### 2.8. ROC Curve Analysis

ROC curve analysis was performed in the testing set to evaluate the discriminatory performance of candidate genes between the CAVD samples and controls. Batch effect correction was applied prior to analysis.

Based on our findings, BAMBI, HAND2, and MYOC were considered inverse association genes, exhibiting higher expression in control samples than in CAVD samples. To maintain consistent directionality in the ROC analysis, the area under the curve (AUC) for these genes was calculated using the negative expression values. ROC curves were generated by plotting sensitivity against 1 − specificity, and the AUC values was calculated to assess discriminative performance. The statistical significance of AUC values was assessed by permutation testing with 1000 iterations. All analyses were performed in R using the pROC package (v1.19.0.1).

### 2.9. scRNA-Seq Analysis of Previously Published Dataset

We obtained previously published raw scRNA-Seq data on the human aortic valve from the BioProject database under the accession number PRJNA562645 [19,20]. Raw sequencing reads were processed using the Cell Ranger toolkit (v8.0.1), which aligned reads to the human reference genome GRCh38 to generate gene–cell count matrices. Seurat (v4.1.1) was used for preprocessing. Cells with fewer than 1000 detected genes were excluded. Gene expression counts were normalized and log-transformed. Highly variable genes were identified, followed by PCA for dimensionality reduction. The top 50 principal components were used to construct a shared nearest neighbor graph, and unsupervised graph-based clustering using Louvain was performed to identify distinct cell populations. Uniform manifold approximation and projection (UMAP) was applied for visualization of cells in two-dimensional space. Cell types were manually annotated based on canonical marker genes. VICs and valve derived stromal cells (VDSCs) were subset and reanalyzed, including the identification of highly variable genes, PCA, re-clustering, and UMAP visualization.

### 2.10. Calcification Score Analysis

Gene set scores were calculated using the AddModuleScore algorithm implemented in the Seurat package with default settings. The calcification level was assessed using a gene signature composed of multiple osteogenic marker genes (Supplemental Table S2).

### 2.11. Pseudotime Analysis

Monocle3 (v1.0.0) was used for single-cell pseudotime analysis. Previously computed results of VICs and VDSCs, including clustering labels and UMAP embeddings, along with raw gene expression counts, were imported into Monocle3. Data preprocessing was performed without additional normalization, and PCA was carried out using 50 dimensions. Cells were then re-clustered within Monocle3, and a principal graph was learned to reconstruct trajectories. The root of the trajectory was defined based on monocle cluster 4, which was exclusively present in the control group and absent in the CAVD group. Cells were then ordered along pseudotime accordingly.

### 2.12. Relative Immune Signatures Analysis

The log_2_(TPM + 1) expression matrix of all samples were uploaded to xCell to estimate relative enrichment scores for immune cell types, which represent inferred immune signatures rather than absolute cell abundances. Only immune signatures showing statistically significant differences between the control and CAVD groups (Wilcoxon rank-sum test, *p* < 0.05) are shown.

### 2.13. Human Samples

Human aortic valve tissues were collected in accordance with relevant guidelines and regulations. The study protocol was approved by the Ethics Committee of Beijing Anzhen Hospital, Capital Medical University (Approval number: KS2019016; Approval date: 2 December 2019), and written informed consent was obtained from all participants.

A total of six cases of aortic valve tissue were collected, of which three cases were calcified aortic valve tissues (Supplemental Table S3). Specimens were taken from CAVD patients who needed aortic valve replacement surgery and the other three cases of control group aortic valve samples were taken from recipients who were clinically diagnosed as aortic dissection and required valve replacement.

### 2.14. Western Blot (WB)

Human aortic valve tissues were cut into small pieces and homogenized in RIPA buffer (1 mL per 100 mg tissue; Solarbio, Beijing, China, Cat#R0010) supplemented with protease and phosphatase inhibitors (1:100; Sigma-Aldrich, St. Louis, MO, USA, Cat#P8340). The lysates were collected into microcentrifuge tubes and centrifuged at 12,000× *g* for 1 min to remove debris. The supernatant was collected, and protein concentration was determined using the bicinchoninic acid (BCA) assay prior to Western blot analysis.

Protein samples were separated on SDS-polyacrylamide gels (Epizyme, Shanghai, China Cat#PG113) and transferred to polyvinylidene fluoride membranes (MilliporeSigma, Billerica, MA, USA, Cat#IPVH00010). The membranes were blocked with 5% Bovine Serum Albumin (Sigma-Aldrich, Billerica, MA, USA, V900933) for 1 h at room temperature. The blocked membranes were incubated overnight at 4 °C with a primary antibody. The membranes were subsequently incubated with a horseradish peroxidase (HRP)-conjugated secondary antibody specific to the primary antibody. Immunoreactive bands were detected with pierce-enhanced chemiluminescence substrate (Solarbia, Beijing, China, Cat#PE0010) and a ChemiDoc MP imaging system (Bio-Rad, Hercules, CA, USA). ImageJ (v1.54h) was used to determine the band density. The primary antibodies used were β-Actin Rabbit antibody (1:1000; Cell Signaling Technology, Danvers, MA, USA, Cat# 4967, rabbit polyclonal), BAMBI antibody (1:1000; Abcam, Cambridge, UK, Cat#ab203070, rabbit polyclonal), HAND2 antibody (1:1000; Abcam, Cambridge, United Kingdom, Cat#ab200040, rabbit monoclonal), and MYOC antibody (1:1000; Abcam, Cambridge, United Kingdom, Cat#ab41552, rabbit polyclonal). The expected sizes of these proteins were 45kDa (β-Actin), 29kDa (BAMBI), 26kDa (HAND2) and 55kDa (MYOC). Goat anti-rabbit HRP-conjugated antibody (1:10,000; Epizyme, Shanghai, China, Cat#LF102) and goat anti-mouse HRP-conjugated antibody (1:5000; Epizyme, Shanghai, China, Cat#LF101) were used as the secondary antibody. The specific signals were identified at the expected molecular weights and used for quantification.

### 2.15. Quantitative Real-Time Polymerase Chain Reaction (qPCR)

Total RNA was extracted from human aortic valve tissues using TRIzol reagent following the manufacturer’s instructions (Invitrogen, Waltham, MA, USA, Cat#15596026). Complementary DNA (cDNA) was synthesized using the PrimeScript RT Reagent Kit (TaKaRa, Beijing, China, Cat#RR036A). Quantitative real-time PCR was performed on optical 96-well plates using a StepOne Real-Time PCR System with SYBR Green PCR reagents (Invitrogen, Waltham, MA, USA, Cat#A25742). Relative gene expression levels were calculated using the Ct (ΔΔCt) method, with results expressed as fold changes relative to the control group. β-Actin was used as the internal reference for normalization. The forward and reverse primer sequences for all genes analyzed by qPCR are listed in Supplemental Table S4.

### 2.16. Statistics

All statistical analyses of public RNA-Seq datasets were performed in R. Differentially expressed genes (DEGs) were identified using the Wald test, and *p* values were adjusted for multiple testing using the BH method. GO and KEGG enrichment analyses were performed using the hypergeometric test with BH correction. The AUC values of feature genes were assessed using permutation tests with 1000 iterations. Relative immune signatures between groups were compared using the Wilcoxon rank-sum test. For clinical samples, qPCR and WB data were analyzed using GraphPad Prism 9 software. Differences between two groups were evaluated using an unpaired Student’s *t*-test. Unless otherwise specified, *p* < 0.05 (or adjusted *p* < 0.05) was considered statistically significant.

## 3. Results

### 3.1. Data Integration and Batch Correction

**Figure 1:**
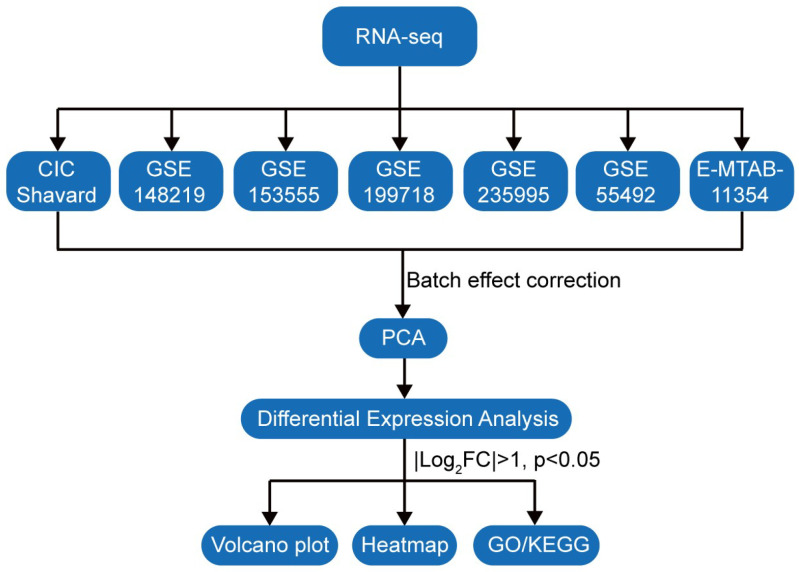
Workflow for identifying differentially expressed genes (DEGs) for CAVD. We first searched the transcriptomic data of CAVD in the GEO database, and finally seven datasets met our criteria. Batch effects were corrected, followed by principal component analysis (PCA) to assess data batch correction. DEGs were then identified, and functional enrichment analyses were performed to explore the associated biological pathways.

**Figure 2:**
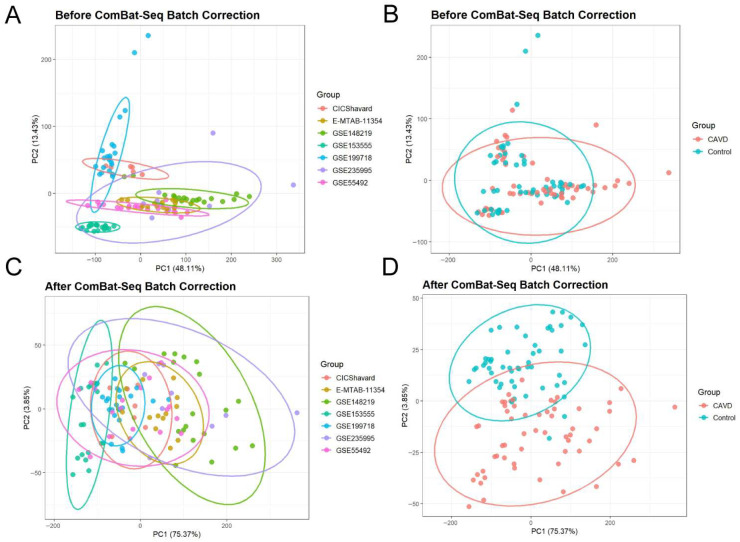
Batch correction of datasets. (**A**,**B**) PCA plots showing the data before batch effect removal by datasets (**A**) and by groups (**B**). (**C**,**D**) PCA plots showing the data after batch effect removal by datasets (**C**) and by groups (**D**). Circles indicate the grouping in each plot.

To identify DEGs associated with CAVD, seven bulk transcriptomic datasets were integrated followed by systematic analyses (Figure 1) [12,13,14,15,16,17,18]. PCA revealed substantial batch effects prior to correction, which could confound biological interpretation (Figure 2A,B). After ComBat-Seq adjustment, samples clustered primarily according to disease status rather than dataset origin (Figure 2C,D), indicating successful batch effect removal and improved data consistency. This corrected dataset enabled reliable downstream analyses, including feature gene selection and functional pathway analysis (Figure 1).

### 3.2. Identification and Functional Enrichment of DEGs in CAVD

**Figure 3:**
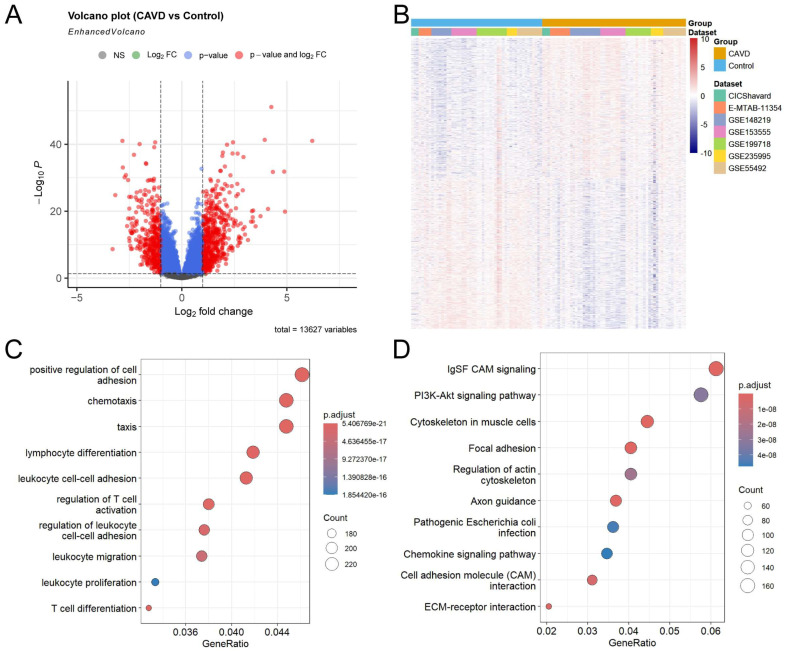
Identification and functional enrichment analysis of DEGs in CAVD. (**A**) Volcano plot showing the overall distribution of DEGs between calcific aortic valve disease (CAVD) and control samples. A total of 391 genes were downregulated (blue) and 510 genes were upregulated (red) in CAVD samples (|log_2_FC| > 1, BH-adjusted p < 0.05). Vertical dotted lines indicate the log_2_FC cutoff (±1), and the horizontal line indicates the *p*-value cutoff (0.05). (**B**) Heatmap showing the mean normalized expression (log_2_(TPM+1)) of DEGs between the CAVD and control samples. (**C**) Gene Ontology (GO) enrichment analysis of DEGs. The count is the number of genes in the set associated with the ontology term. *p* values were calculated based on the cumulative hypergeometric distribution with BH correction. The top ten significantly enriched terms are shown. (**D**) Kyoto Encyclopedia of Genes and Genomes (KEGG) pathway enrichment analysis. The count is the number of genes in the set associated with the pathway. *p* values were calculated based on the cumulative hypergeometric distribution with BH correction. The top ten significantly enriched pathways are shown.

In order to characterize transcriptional differences between CAVD and control samples, DEGs were identified from batch-corrected count data, ensuring unbiased estimation of group-specific expression differences. A total of 510 upregulated and 391 downregulated genes were obtained using a cutoff of |log_2_FC| > 1 and BH-adjusted *p* < 0.05 (Figure 3A). The clear and consistent separation between CAVD and control samples was demonstrated, indicating distinct transcriptomic profiles (Figure 3B). To investigate the biological relevance of these DEGs, GO and KEGG enrichment analyses were performed. These genes were predominantly enriched in pathways related to IgSF cell adhesion molecule (CAM) signaling, PI3K-Akt signaling, cytoskeleton in muscle cells, positive regulation of cell adhesion, chemotaxis, taxis and lymphocyte differentiation, suggesting that immune activation, cell migration, and tissue remodeling are key processes in CAVD (Figure 3C,D).

### 3.3. Feature Selection for CAVD

The DEG analysis provided a comprehensive overview of transcriptional alterations associated with CAVD using all samples after unified batch correction. To identify the most informative features associated with CAVD, machine learning-based feature selection was subsequently performed.

**Figure 4:**
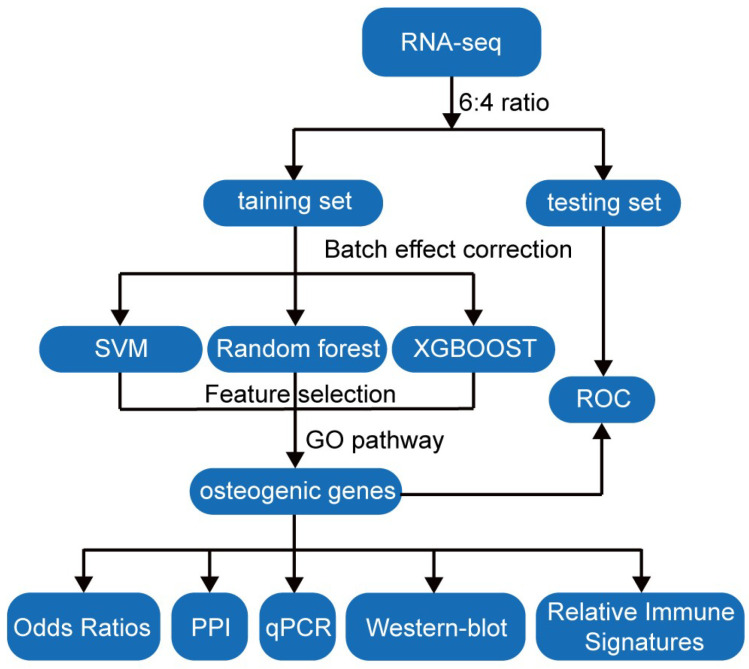
Workflow of machine learning–based feature selection. The datasets were stratified into training and testing sets at a 6:4 ratio with balanced class distribution. Batch effect correction was performed separately in the training and testing sets to minimize technical variability. Three machine learning algorithms—support vector machine with linear kernel (SVM-Linear), random forest (RF), and extreme gradient boosting (XGBoost)—were applied in the training set to identify osteogenesis-related genes. The discriminative performance of the candidate genes was subsequently validated in the testing set. The candidate genes were further evaluated through odds ratio (OR) analysis, protein–protein interaction (PPI) network construction, quantitative real-time polymerase chain reaction (qPCR) and Western blot (WB) validation, and relative immune signatures analysis.

Specifically, samples were stratified into training and testing sets at a 6:4 ratio with balanced class distribution, and batch effect correction was re-applied within the training set to remove technical variability and prevent information leakage. Afterwards, three machine learning algorithms—SVM-Linear, RF, and XGBoost—were employed for feature selection (Figure 4).

**Figure 5:**
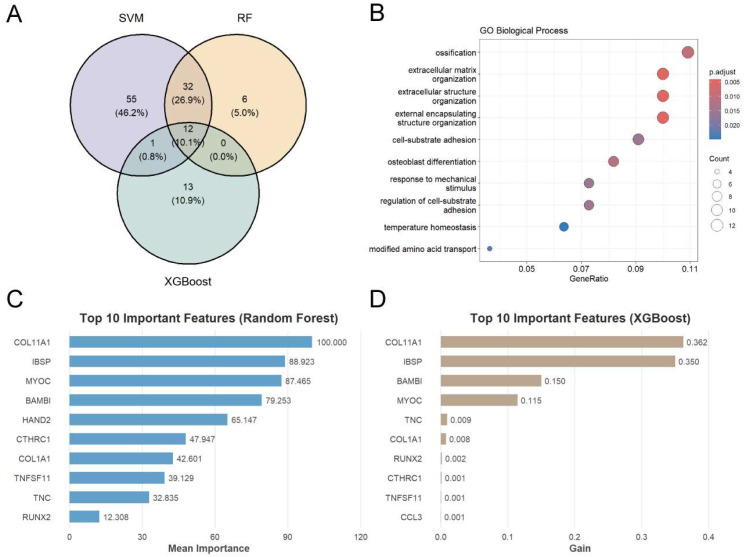
Feature selection and functional enrichment analysis of candidate genes associated with CAVD. (**A**) Venn diagram showing the overlap of feature genes identified by three machine learning algorithms: SVM-Linear, RF, and XGBoost. The numbers indicate the count of genes selected by each method. (**B**) GO enrichment analysis of the union of feature genes identified by the three algorithms. The count is the number of genes in the set associated with the ontology term. *p* values were calculated based on the cumulative hypergeometric distribution with BH correction. The top ten significantly enriched terms are shown. (**C**) Ranking of genes involved in the ossification pathway based on feature importance scores calculated by RF. The *x*-axis represents the importance score, with higher values indicating greater contribution to the model. The top ten significantly enriched genes are shown. (**D**) Ranking of genes involved in the ossification pathway based on feature importance scores calculated by XGBoost. The *x*-axis represents the gain, reflecting the contribution of each gene to the model’s predictive performance. The top ten significantly enriched genes are shown.

To explore the potential biological functions of the candidate genes associated with CAVD, the union of genes identified by the three algorithms was subjected to GO functional enrichment analysis (Figure 5A). The analysis revealed that these genes were significantly enriched in biological processes related to ossification, extracellular matrix organization, extracellular structure organization, external encapsulating structure organization, cell–substrate adhesion, osteoblast differentiation, and response to mechanical stimulus (Figure 5B).

We then focused on the ossification pathway by ranking the enriched genes according to their importance using RF and XGBoost, as these tree-based models provide explicit feature importance scores, whereas SVM primarily serves as a classifier (Figure 5C,D). Interestingly, COL11A1, BAMBI, IBSP, CTHRC1, TNC, MYOC, TNFSF11, RUNX2, HAND2, COL1A1, and CCL3 ranked among the top genes in both algorithms. COL11A1, a member of the collagen protein family and a structural component of the ECM, has been reported to be upregulated in calcified aortic valve tissues [13,21,22]. CTHRC1 is associated with the TGF-β/BMP signaling pathways, which are critical for the activation of VICs and ECM remodeling [23]. TNC is a matricellular protein with reportedly strong deposition in calcified regions, which may contribute to ECM remodeling, thereby promoting valve calcification [24]. TNFSF11 is involved in bone formation and resorption balance, and it is upregulated in calcified aortic valve tissues, where it can stimulate VICs to undergo osteogenic differentiation [25,26]. In addition, previous studies have reported that CCL3 expression is elevated in calcified aortic valves, with immunofluorescence staining demonstrating co-localization with infiltrating macrophages, indicating a potential role in valvular inflammation and calcification [27]. Both RUNX2 and IBSP are regarded as molecular markers of calcified aortic valves and act as promoters of mineralization [28,29,30,31,32]. In contrast, the biological role of BAMBI, HAND2, and MYOC in valvular calcification remains unclear. Therefore, these three genes were identified as novel candidate genes associated with CAVD in this study.

### 3.4. Association of Candidate Genes with CAVD and Functional Interactions

**Figure 6:**
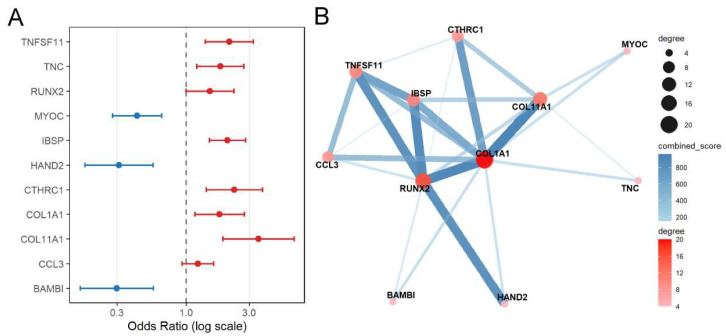
Forest plot of univariate logistic regression results for the candidate genes and the corresponding PPI network. (**A**) Forest plot showing odds ratios (ORs) and 95% confidence intervals (CIs) from univariate logistic regression for each candidate gene. ORs represent the change in odds of CAVD per one-unit increase in log_2_-transformed gene expression (log_2_(count + 1)). Points indicate ORs, horizontal bars indicate 95% CIs, and the dashed line represents OR = 1. (**B**) PPI network of candidate genes, constructed using the STRING database. Nodes represent proteins, and edges represent predicted or experimentally validated interactions. Both node size and color are proportional to the degree (number of interactions) of each protein, with larger and darker nodes indicating higher connectivity. Edge thickness indicates the confidence score of the interaction, with thicker edges representing higher-confidence interactions.

To investigate the potential association of candidate osteogenic genes with CAVD, we performed univariate logistic regression analysis. COL11A1 (OR = 3.52, 95% CI: 1.89–6.53), IBSP (OR = 2.05, 95% CI: 1.50–2.81), CTHRC1 (OR = 2.31, 95% CI: 1.42–3.76), TNC (OR = 1.81, 95% CI: 1.20–2.72), TNFSF11 (OR = 2.11, 95% CI:1.39–3.21), RUNX2 (OR = 1.51, 95% CI: 1.00–2.29), and COL1A1 (OR = 1.78, 95% CI: 1.16–2.75) exhibited ORs greater than 1, indicating a positive association with CAVD risk, which is consistent with the previous descriptions (Figure 6A). In contrast, BAMBI (OR = 0.30, 95% CI: 0.16–0.56), HAND2 (OR = 0.31, 95% CI: 0.17–0.56), and MYOC (OR = 0.42, 95% CI: 0.28–0.65) had ORs less than 1, indicating a possible inverse relationship with CAVD (Figure 6A).

Furthermore, to characterize the functional relationships among these candidate genes, a PPI network was constructed using the STRING database (Figure 6B). BAMBI, HAND2, and MYOC were located at the periphery of the PPI network, with relatively smaller node sizes, indicating fewer direct interactions with other ossification-related genes. This suggests that their roles in the ossification process are less well-characterized, highlighting them as novel candidate genes contributing to CAVD that merit further investigation.

### 3.5. Performance Evaluation of Novel Candidate Genes by ROC Analysis

**Figure 7:**
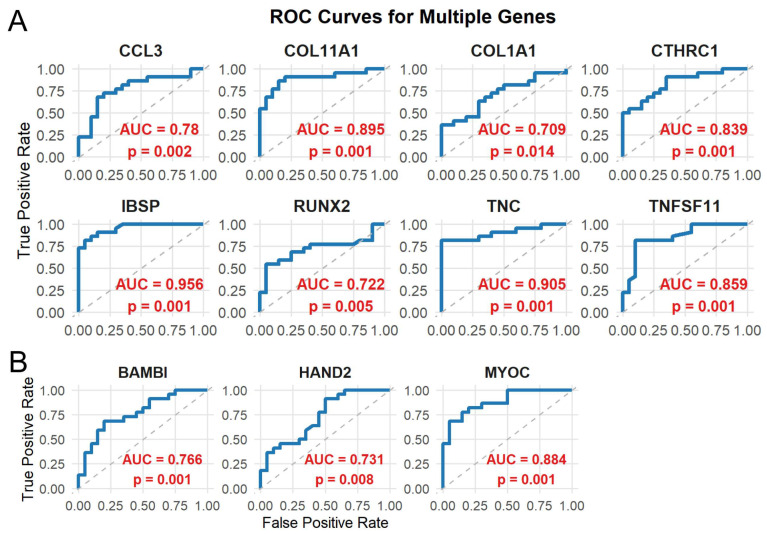
Receiver operating characteristic (ROC) curve analysis of candidate genes for discriminating CAVD from control samples. (**A**) ROC curves of risk genes with higher expression in CAVD, indicative of potential pathogenic roles. (**B**) ROC curves of inverse association genes that were downregulated in CAVD. For each gene, the area under the curve (AUC) is shown to assess its discriminatory ability between CAVD and control samples, and statistical significance was determined by permutation testing (1000 iterations). The diagonal dashed line at 45° represents the line of no discrimination (AUC = 0.5).

**Figure 8:**
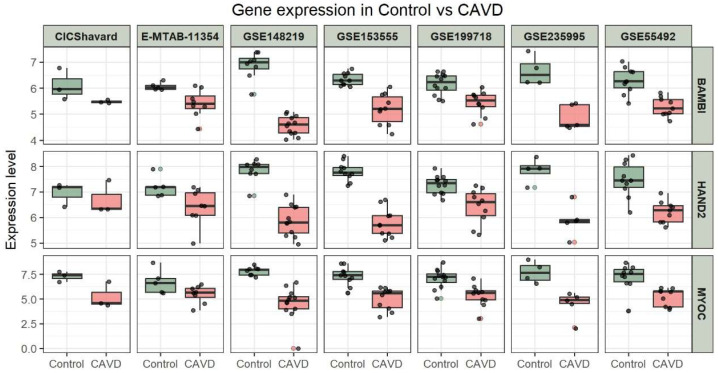
Expression of novel candidate genes in CAVD and control samples. Gene expression profile (log_2_(TPM+1)) in CAVD and control samples for BAMBI, HAND2, and MYOC genes. Box plots represent the interquartile range (IQR), with the median shown as a horizontal line inside the box. The whiskers extend to the minimum and maximum values excluding outliers. Each dot represents a sample.

ROC analysis was performed for each candidate gene in the testing set to evaluate their ability to discriminate CAVD from control samples. All genes demonstrated favorable discriminative performance (Figure 7). While several CAVD risk genes have been extensively documented in the literature, the three inversely associated genes BAMBI, HAND2, and MYOC remain relatively unexplored. Therefore, subsequent analyses and experimental validations were focused on these three novel candidate genes. Expression profiling revealed that all three genes were significantly higher in the control samples than in the CAVD samples, consistent with their negative association with CAVD (Figure 8).

### 3.6. scRNA-Seq Analysis of BAMBI, HAND2, and MYOC

**Figure 9:**
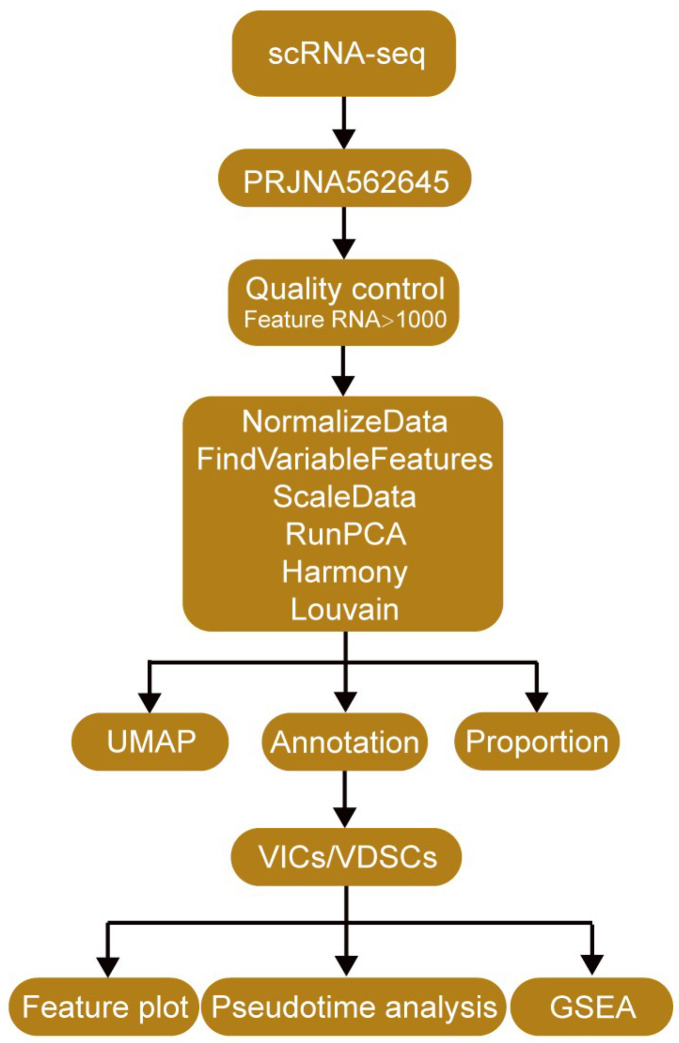
Workflow of single-cell RNA-sequencing (scRNA-Seq) analysis of valve interstitial cells (VICs) and valve derived stromal cells (VDSCs). Raw scRNA-Seq data from human aortic valves were processed using Cell Ranger and Seurat. After preprocessing, unsupervised clustering and UMAP were applied for dimensionality reduction and visualization of cell populations. VICs and VDSCs were then identified based on canonical marker gene expression and extracted for downstream analyses. Feature plots were generated to visualize the expression patterns of novel candidate genes in VICs and VDSCs. Gene Set Enrichment Analysis (GSEA) and pseudotime trajectory analysis were subsequently performed to investigate functional pathway enrichment and dynamic transcriptional changes during VIC-to-VDSC transition.

During osteogenic transdifferentiation, VICs transform to a novel subpopulation, VDSCs [20]. To investigate gene expression changes associated with this process, we analyzed previously published human aortic scRNA-Seq data [19,20], focusing on VICs and VDSCs (Figure 9).

**Figure 10:**
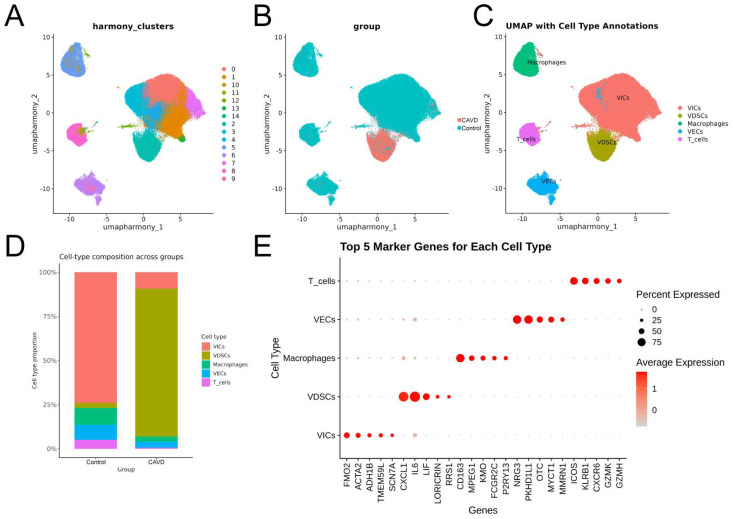
UMAP visualization and characterization of cell populations in human aortic tissue. (**A**–**C**) UMAP plots of human aortic tissue showing distinct cell clusters (**A**), sample groups (**B**), and annotated cell types (**C**). Cell types were annotated as follows: VICs (clusters 0, 1, 3, 4, 7, 12, 13, and 14), VDSCs (cluster 2), macrophages (clusters 5 and 10), VECs (clusters 6 and 9), and T cells (clusters 8 and 11). Each point represents a single cell. (**D**) Relative proportions of major cell types in each group. (**E**) Bubble plot showing selected marker genes of each cluster.

UMAP visualization demonstrated distinct cell clusters, sample groups, and cell types (Figure 10A–C). The relative proportions of major cell types were quantified, and canonical marker genes were used to annotate each cluster (Figure 10D,E).

**Figure 11:**
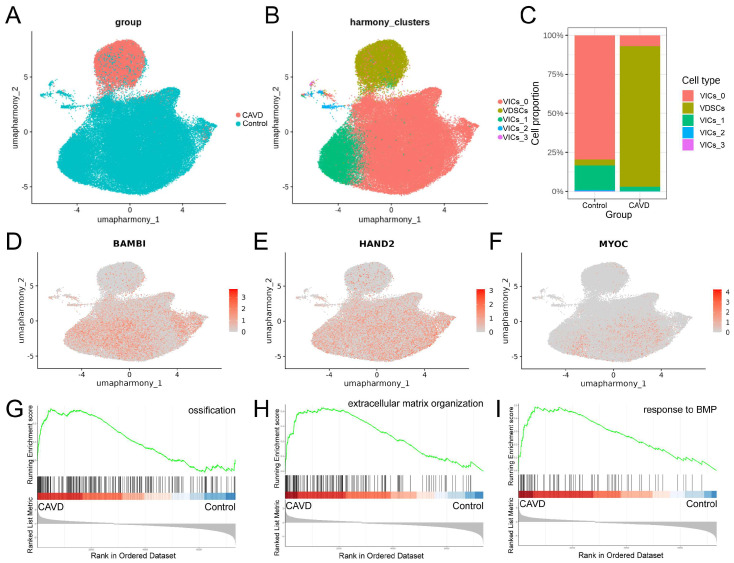
Single-cell expression of BAMBI, HAND2, and MYOC and pathway enrichment analysis in CAVD and control samples. (**A**,**B**) UMAP plots of VICs/VDSCs after re-clustering. Each point represents a single cell and is colored according to its sample group (**A**) or cluster label that indicates major VICs/VDSCs subtypes (**B**). (**C**) Relative proportions of major VIC/VDSC subtypes in each group. The *y*-axis represents the proportion of cells of each subtype, and the *x*-axis represents sample groups. (**D**–**F**) UMAP plots showing the expression of BAMBI (**D**), HAND2 (**E**), and MYOC (**F**) across VICs/VDSCs. Expression levels are indicated by color intensity, with darker colors representing higher expression. (**G**–**I**) GSEA ridge plots comparing CAVD and control samples. Each ridgeline represents a pathway, and the height corresponds to the normalized enrichment score (NES). The green curve shows the enrichment score (ES) of the gene set across the ranked gene list, black tick marks indicate gene positions, and the gradient at the bottom represents the ranking metric (log_2_FC), with left (red) indicating genes upregulated in CAVD versus control and right (blue) indicating genes downregulated in CAVD versus control.

The switch of VICs from a quiescent state to an osteogenic state (VDSCs) is the key event driving aortic valve calcification. To investigate this, we re-clustered VICs and VDSCs, and identified five distinct subtypes (Figure 11A,B). The relative proportions of major VICs/VDSCs types were quantified (Figure 11C). Consistent with the bulk RNA-Seq results, BAMBI, HAND2, and MYOC were predominantly expressed in the control group (VICs) compared with the CAVD group (VDSCs) (Figure 11D–F). This observation reinforces the negative association of these genes with CAVD and suggests that their differential expression is preserved at the single-cell level. Gene Set Enrichment Analysis (GSEA) revealed distinct enrichment patterns between CAVD and control samples at the pathway level. Pathways related to ossification, extracellular matrix organization, and response to BMP were strongly upregulated in CAVD, underscoring the heightened activation of osteogenic and ECM-remodeling processes in calcified aortic valve tissues (Figure 11G–I).

### 3.7. Pseudotime Analysis of VICs and VDSCs

**Figure 12:**
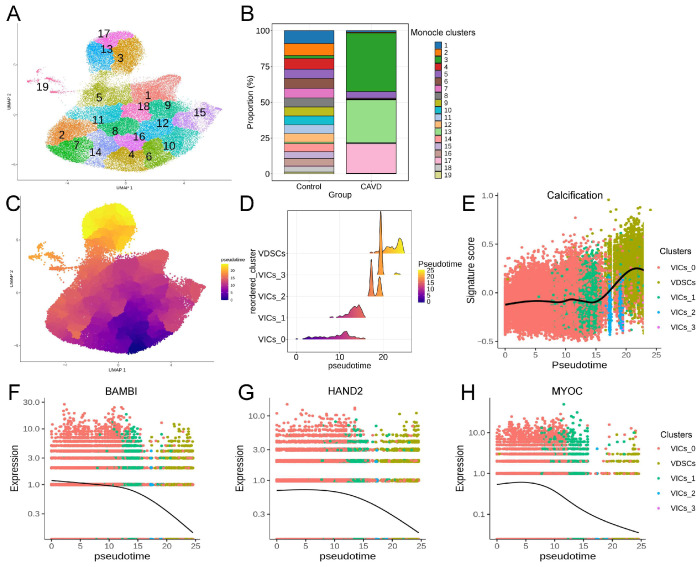
Pseudotime analysis and expression dynamics of novel candidate genes in VICs/VDSCs. (**A**) Re-clustering of VICs/VDSCs by Monocle3. Each point represents a single cell and is colored according to its Monocle3 cluster identity, with cluster numbers recalculated by Monocle3. (**B**) Relative proportions of monocle clusters in each group. The *y*-axis represents the proportion of cells of each monocle cluster, and the *x*-axis represents sample groups. (**C**) UMAP visualization of VICs/VDSCs pseudotime scores. (**D**) Ridge plot showing VIC/VDSC cluster distribution along pseudotime, colored by pseudotime scores. (**E**) The score of calcification signature along the pseudotime trajectory. (**F**–**H**) Expression of BAMBI (**F**), HAND2 (**G**), and MYOC (**H**) along the pseudotime trajectory.

To investigate the dynamic transcriptional changes underlying VIC differentiation in CAVD, we performed pseudotime analysis using Monocle3. VICs and VDSCs were re-clustered, resulting in the identification of multiple Monocle-defined clusters (Figure 12A). Examination of monocle cluster distributions in the control and CAVD samples showed that monocle cluster 4, which corresponds to a subset of VIC_0, was exclusively present in the CAVD samples and absent in the controls (Figure 12B). In contrast, monocle clusters 3, 13, and 17 (VDSCs) were significantly enriched in CAVD samples, reflecting an osteogenic VDSC phenotype. Based on these observations, monocle cluster 4 was designated as the root/quiescent state. The reconstructed pseudotime trajectory revealed a continuous progression from quiescent VICs (VIC_0) toward osteogenic VDSCs (Figure 12C,D). Consistently, the calcification score that was characterized by a signature of multiple osteogenic marker genes (Supplemental Table S2), gradually increased along the trajectory, indicating the progressive acquisition of osteogenic features (Figure 12E).

Importantly, the expression of BAMBI, HAND2, and MYOC progressively decreased along the pseudotime trajectory (Figure 12F–H), suggesting that their downregulation may precede or coincide with osteogenic commitment. Overall, these results suggest a transcriptionally inferred trajectory from quiescent VICs to osteogenic VDSCs in CAVD, with the downregulation of BAMBI, HAND2 and MYOC potentially associated with this transition.

### 3.8. Relative Immune Signatures Analysis of All Integrated RNA-Seq Datasets

**Figure 13:**
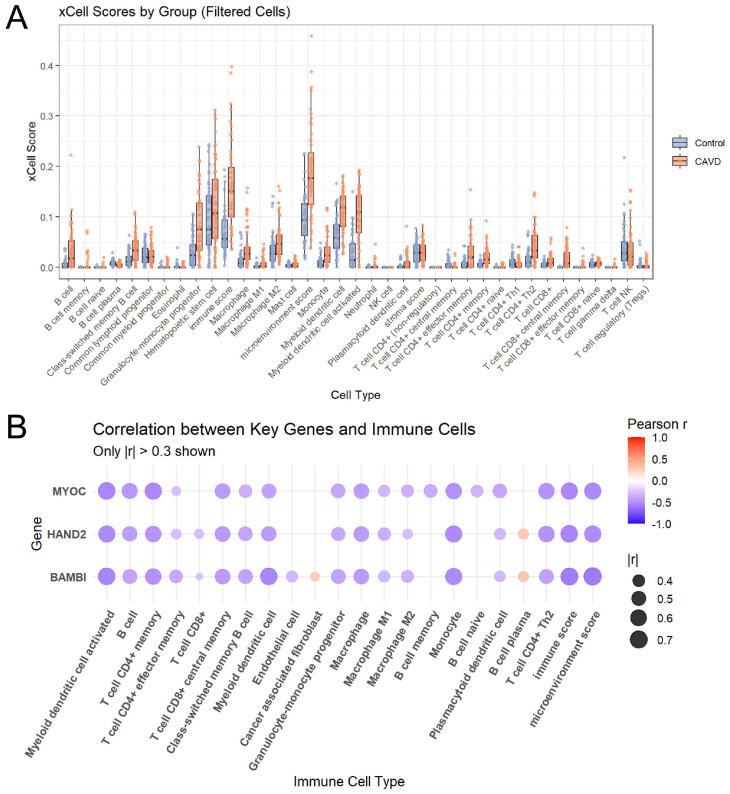
Analysis of Relative Immune Signatures by xCell. (**A**) Box plots showing xCell-derived relative immune signatures for CAVD (orange) and control samples (blue). Differences between groups were assessed using the Wilcoxon test, and only groups with *p* < 0.05 are shown. (**B**) The bubble plot showing correlations between novel candidate genes (BAMBI, HAND2, and MYOC) and relative immune signatures. Only results with the absolute value of correlation coefficient (r) greater than 0.3 are shown.

To explore the immune landscape in CAVD, we applied the xCell algorithm to estimate relative immune signatures across all integrated RNA-Seq datasets. As a result, relative immune signatures of several myeloid and lymphoid differed between the control and CAVD samples, which may reflect changes in the local immune microenvironment during disease progression (Figure 13A).

We then examined the correlations between novel candidate genes (BAMBI, HAND2, and MYOC) and relative immune signatures. These genes displayed moderate negative correlations with multiple immune cell types (Figure 13B). Specifically, BAMBI, HAND2, and MYOC were negatively correlated with the overall immune score and microenvironment score (Pearson r < −0.3). Importantly, as these genes are primarily expressed in non-immune cells, such as valvular cells, these correlations likely reflect indirect associations, such as tissue remodeling or microenvironmental changes, rather than the direct modulation of immune cells.

### 3.9. Validation of Novel Candidate Gene Expression in Calcified Aortic Valve Tissues

**Figure 14:**
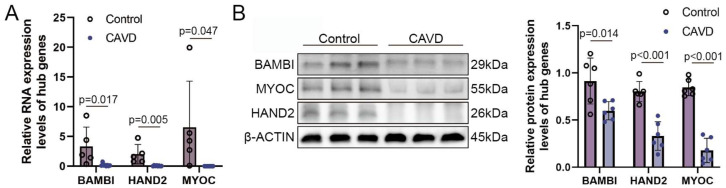
Validation of Novel Candidate Genes Expression in Human Calcified Aortic Valve Tissues. (**A**) Quantification of mRNA expression by qPCR. Relative transcript levels of BAMBI, HAND2, and MYOC in human valve tissues from control and CAVD groups were calculated using the 2^−ΔΔCt^ method and normalized to β-actin. *p* values were calculated using unpaired Student’s *t*-tests. (**B**) Representative immunoblots and densitometric data showing the expression levels of BAMBI, HAND2, and MYOC in CAVD samples compared to normal control tissues. *p* values were calculated using unpaired Student’s *t*-tests.

To further validate the transcriptional alterations identified in our sequencing analyses, we examined the expression of BAMBI, HAND2, and MYOC in calcified and non-calcified aortic valve tissues. qPCR analysis revealed that the mRNA levels of all three genes were significantly decreased in calcified valves compared with non-calcified controls (Figure 14A). These results were highly consistent with the transcriptomic findings, confirming the robustness of our sequencing-derived gene expression patterns.

To determine whether these changes in transcription were reflected at the protein level, we next performed Western blot analysis. As a result, the protein expression levels of BAMBI, HAND2, and MYOC were markedly reduced in calcified aortic valve tissues relative to non-calcified controls (Figure 14B). The full uncropped gels are provided in Supplemental Material File S2. The concordance between mRNA and protein expression further supports the biological relevance of these genes and reinforces their potential involvement in the pathological progression of aortic valve calcification.

## 4. Discussion

CAVD has emerged as a major clinical concern worldwide, particularly among patients with chronic conditions such as chronic kidney disease, diabetes mellitus, and coronary artery disease. CAVD is highly prevalent and often progresses to aortic stenosis, resulting in heart failure and, ultimately, increased mortality [10,33,34]. The etiology of CAVD is complex, involving multiple factors. With aging, valve growth slows and the regenerative capacity of valvular cells becomes insufficient to repair injury [35]. Disturbances in lipid metabolism, sustained mechanical stress, and chronic immune and inflammatory activation can lead to endothelial dysfunction and extracellular matrix remodeling, ultimately causing valvular injury and calcific deposition [4,36,37]. Currently, no pharmacological therapy has been proven to halt CAVD progression, and aortic valve replacement remains the only effective treatment [38,39].

In this study, we identified a set of DEGs between the CAVD and control samples. Subsequent GO and KEGG enrichment analyses revealed that these genes were significantly associated with IgSF CAM signaling, PI3K-Akt signaling pathway, cytoskeleton in muscle cells, positive regulation of cell adhesion, chemotaxis, taxis and lymphocyte differentiation pathways, highlighting potential inflammatory molecular, cell migration, and tissue remodeling driving calcific aortic valve disease. These findings are consistent with previous reports [2,10,37,40].

To further investigate the regulatory network and molecular mechanisms underlying CAVD, we applied three complementary machine learning approaches—SVM-Linear, RF, and XGBoost—to screen for candidate genes. By integrating these methods, we prioritized genes that are potentially pivotal in mediating ossification, extracellular matrix organization, extracellular structure organization, external encapsulating structure organization, cell–substrate adhesion, osteoblast differentiation, and response to mechanical stimulus in the calcifying aortic valve. We specifically focused on genes enriched in ossification pathways, as these may be involved in valvular calcification and disease progression. By applying RF and XGBoost algorithms, we prioritized a set of candidate genes, including COL11A1, BAMBI, IBSP, CTHRC1, TNC, MYOC, TNFSF11, RUNX2, HAND2, COL1A1, and CCL3. Furthermore, integration of PPI network analysis with ROC evaluation highlighted BAMBI, HAND2, and MYOC—genes rarely reported in the context of aortic valve calcification—as novel candidate genes showing potential discriminative ability. Accordingly, we directed our downstream analyses toward these three genes as novel candidates. These three genes exhibited significantly higher expression in the control samples relative to the CAVD samples, and this pattern was consistently validated using sc-RNA-Seq data and calcified aortic valve tissues. Consistent with these observations, single-cell pseudotime analysis demonstrated that the downregulation of BAMBI, HAND2, and MYOC may occur prior to the osteogenic commitment of VICs. These results provide support for the hypothesis that the loss of BAMBI/HAND2/MYOC may be involved in the VIC-to-osteogenic transition rather than a bystander effect.

BAMBI is a pseudoreceptor of the TGF-β/BMP signaling pathway, thereby functioning as a decoy receptor to inhibit downstream TGF-β/BMP signaling [41,42,43,44]. Previous studies suggest that reduced BAMBI expression promotes osteogenic differentiation, likely by relieving its inhibitory effect on BMP signaling [45,46]. HAND2 regulates the differentiation of mesenchymal cells and has been reported to inhibit osteogenic transdifferentiation by physically interacting with and suppressing the DNA binding activity of RUNX2, a master regulator of osteoblast differentiation [47,48]. Moreover, Hand2 hypomorphic mice exhibit premature ossification associated with upregulated Runx2 expression [49]. In addition, the overexpression of Hand2 in limb-bud/chondrogenic models suppresses chondrocyte maturation and endochondral ossification [50]. MYOC (myocilin) is an immunoglobulin-like protein expressed in mesenchymal stem cells and cardiovascular tissues, contributing to extracellular matrix organization and cellular homeostasis, with loss or dysfunction potentially facilitating aberrant osteogenic differentiation [51,52]. In this study, BAMBI, HAND2, and MYOC expression was higher in the control samples than in the CAVD samples, indicating a potential inverse association of these genes with valvular calcification. Taken together, these findings suggest that BAMBI, HAND2, and MYOC may represent promising candidate biomarkers and potential targets for further investigation in CAVD.

To explore the immune microenvironment in CAVD, we applied the xCell algorithm to estimate relative immune cell signatures across integrated RNA-Seq datasets. All examined immune cell types showed higher inferred proportions in CAVD samples compared with the controls. These findings may reflect alterations in the local immune microenvironment and need further validation. Furthermore, BAMBI, HAND2, and MYOC showed moderate correlations with several relative immune signatures. However, these genes are predominantly expressed in non-immune cells, such as valve interstitial or fibroblast-like cells. Therefore, these associations likely reflect indirect effects related to tissue remodeling or microenvironmental changes rather than direct immunomodulatory activity.

Several limitations of this study should be acknowledged. First, the public transcriptomic datasets used in this analysis lacked detailed clinical information, such as echocardiographic parameters like mean transvalvular gradient and valve area. In addition, although human aortic valve tissues were used for Western blot and qPCR validation, the small sample size (three CAVD and three control samples) limited statistical power and prevented stratified analyses according to disease stage or valve hemodynamic severity. Second, although BAMBI, HAND2, and MYOC demonstrated moderate to relatively high discriminative performance, these findings should be considered exploratory due to potential performance inflation and the use of similar publicly available datasets which are exploratory in nature rather than directly applicable to clinical diagnostics. Third, while these genes were inversely associated with osteogenic markers, their causal roles in valvular calcification were not directly tested and require functional perturbation experiments such as knockdown or overexpression in valve interstitial cells. Finally, because xCell provides inferred rather than directly measured immune cell abundances, and the fibrotic nature of valve tissue may bias deconvolution, these results should be regarded as hypothesis-generating and warrant further validation using spatial transcriptomics or immunohistochemistry.

Despite these limitations, our findings provide a basis for future clinical and translational research. The identified gene signatures may serve as candidate biomarkers for CAVD progression and require validation in larger, well-characterized cohorts. They may also offer potential therapeutic targets, with strategies aimed at preserving the quiescent VIC phenotype or inhibiting early osteogenic commitment. Further mechanistic studies could inform the development of pharmacological or gene-based interventions.

## 5. Conclusions

This study indicates that BAMBI, HAND2, and MYOC are significantly downregulated in CAVD and are inversely associated with inferred relative immune signatures. These findings suggest a potential link between these genes and tissue remodeling or immune microenvironmental changes, providing a foundation for future mechanistic studies to investigate their roles in valvular calcification and microenvironmental regulation.

## Data Availability

The bulk RNA-Seq datasets analyzed in this study were obtained from publicly available repositories, including the CICS MultiOmics database (Brigham and Women’s Hospital, Harvard Medical School), the Gene Expression Omnibus (GEO; National Center for Biotechnology Information, USA), and the EMBL-EBI Expression Atlas. The GEO and EMBL-EBI datasets were accessed under accession numbers GSE148219, GSE153555, GSE199718, GSE235995, GSE55492, and E-MTAB-11354. The dataset derived from the CICS MultiOmics database is referred to in this study as “CICSharvard” for convenience. In addition, the single-cell RNA sequencing dataset was obtained from the BioProject database under accession number PRJNA562645.

## Supplementary Materials

The following supporting information can be downloaded at: https://www.mdpi.com/article/10.3390/genes17020246/s1. Supplemental Material File S1: Table S1: Basic information of the transcriptome datasets included in this study; Table S2: Gene list for calcification score calculation; Table S3: Clinical characteristics of individual samples; Table S4: Primer sequences used for quantitative PCR (qPCR). Supplemental Material File S2: The Full Uncropped Gels.

## Abbreviations

CAVD	Calcified aortic valve disease
TAVR	Surgical and transcatheter aortic valve replacement
VICs	Valvular interstitial cells
VDSCs	Valve derived stromal cells
ECM	Extracellular matrix
DEGs	Differentially expressed genes
GO	Gene Ontology
KEGG	Kyoto Encyclopedia of Genes and Genomes
SVM-Linear	Support vector machine with linear kernel
RF	Random forest
XGBoost	Extreme gradient boosting
PPI	Protein–protein interaction
ROC	Receiver Operating Characteristic
AUC	Area under the curve
PCA	Principal component analysis
GSEA	Gene Set Enrichment Analysis
WB	Western blot analysis
qPCR	Quantitative real-time polymerase chain reaction
