# Supplementary material for: Integrated Transcriptomic Analysis Identifies Novel Candidate Genes Associated with Calcific Aortic Valve Disease
Source: Genes (Basel). 2026 Feb 20;17(2):246. doi: 10.3390/genes17020246 (PMC12941080; doi:10.3390/genes17020246)
Supplement: Supplementary file 1 [file genes-17-00246-s001.zip › Supplemental meterial S1.pdf]

SUPPLEMENTAL MATERIAL S1

Table S1. Basic information of the transcriptome datasets included in this study.

Datasets	Participants	Year
CICShavard	3CAVD and 3control	2018
GSE148219	12CAVD and 8control	2021
GSE153555	10CAVD and 10control	2020
GSE199718	10CAVD and 12control	2022
GSE235995	5CAVD and 4control	2023
GSE55492	9CAVD and 10control	2015
E-MTAB-11354	8CAVD and 5control	2022

The table summarizes the GEO accession IDs, participant numbers, and publication years of the datasets included in this study.

Table S2. Gene list for calcification score calculation.

Genes
THBS2
IBSP
MSX2
RUNX2
BMP2
IL6
DLX5
MSX1
ATF4
TNF
CTNNB1
MMP10

MMP2
WNT11
SLC20A1

Table S3. Clinical characteristics of individual samples.

Patients	Sex	Age	Velocity (mm/s)	Pressure (mmHg)	Hypertension	Diabetes
Control 1	Male	55	2360	22	1	1
Control 2	Male	48	2140	18	1	0
Control 3	Male	62	2020	16	1	1
CAVD 1	Male	37	3490	30	0	0
CAVD 2	Female	66	4120	52	0	0
CAVD 3	Female	61	4630	86	0	0

Table S4. Primer sequences used for quantitative PCR (qPCR).

Gene	Primer	Sequence (5’- 3’)
ACTB	Forward	CACCATTGGCAATGAGCGGTTC
	Reverse	AGGTCTTTGCGGATGTCCACGT
BAMBI	Forward	TACAGAGGGCTGCACGATGTTC
	Reverse	AAGTCAGCTCCTGCACCTTGGT
HAND2	Forward	GGCAGAGATCAAGAAGACCGAC
	Reverse	CGGCCTTTGGTTTTCTTGTCGTT
MYOC	Forward	CCATCTGGCTATCTCAGGAGTG
	Reverse	GCATCCACACACCATACTTGC

This table lists the forward and reverse primer sequences for all genes analyzed by qPCR in this study.
