# Supplementary material for: Integrated Transcriptomic Analysis Identifies Novel Candidate Genes Associated with Calcific Aortic Valve Disease
Source: Genes (Basel). 2026 Feb 20;17(2):246. doi: 10.3390/genes17020246 (PMC12941080; doi:10.3390/genes17020246)

SUPPLEMENTAL MATERIAL S2

The Full Uncropped Gels

indicates the data shown in the manuscript.

HAND2 (26 kDa)

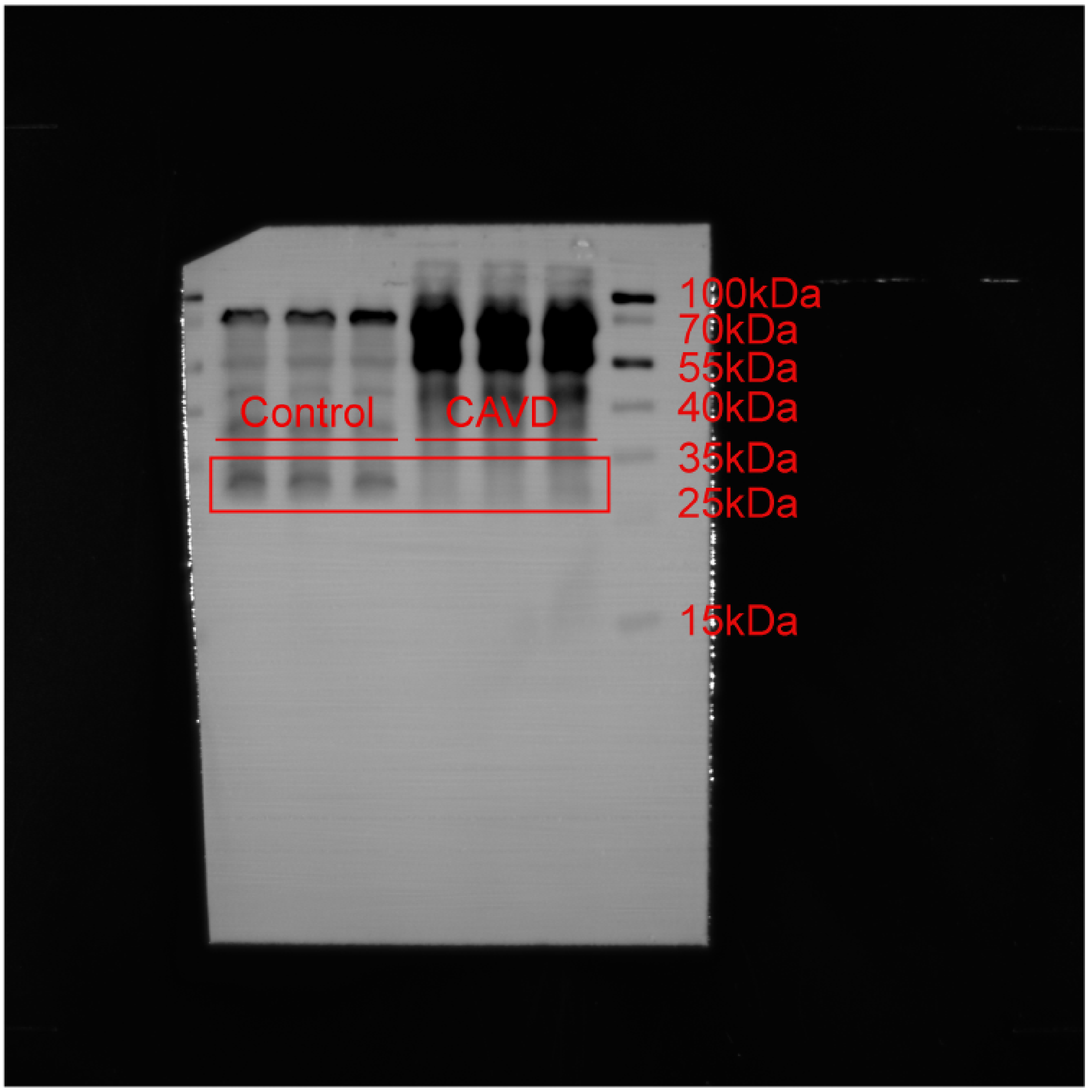

BAMBI (29kDa)

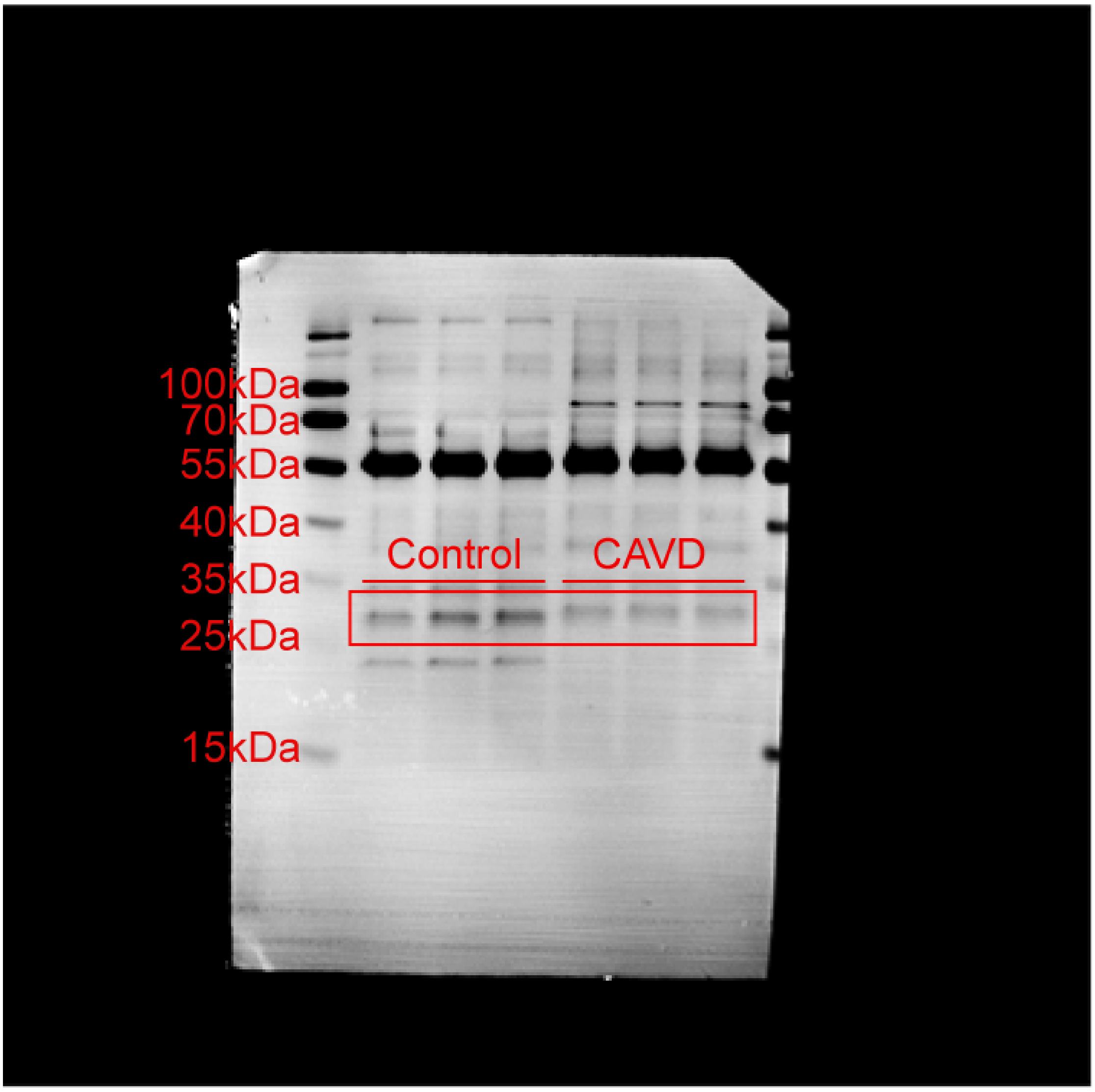

B-ACTIN (45kDa)

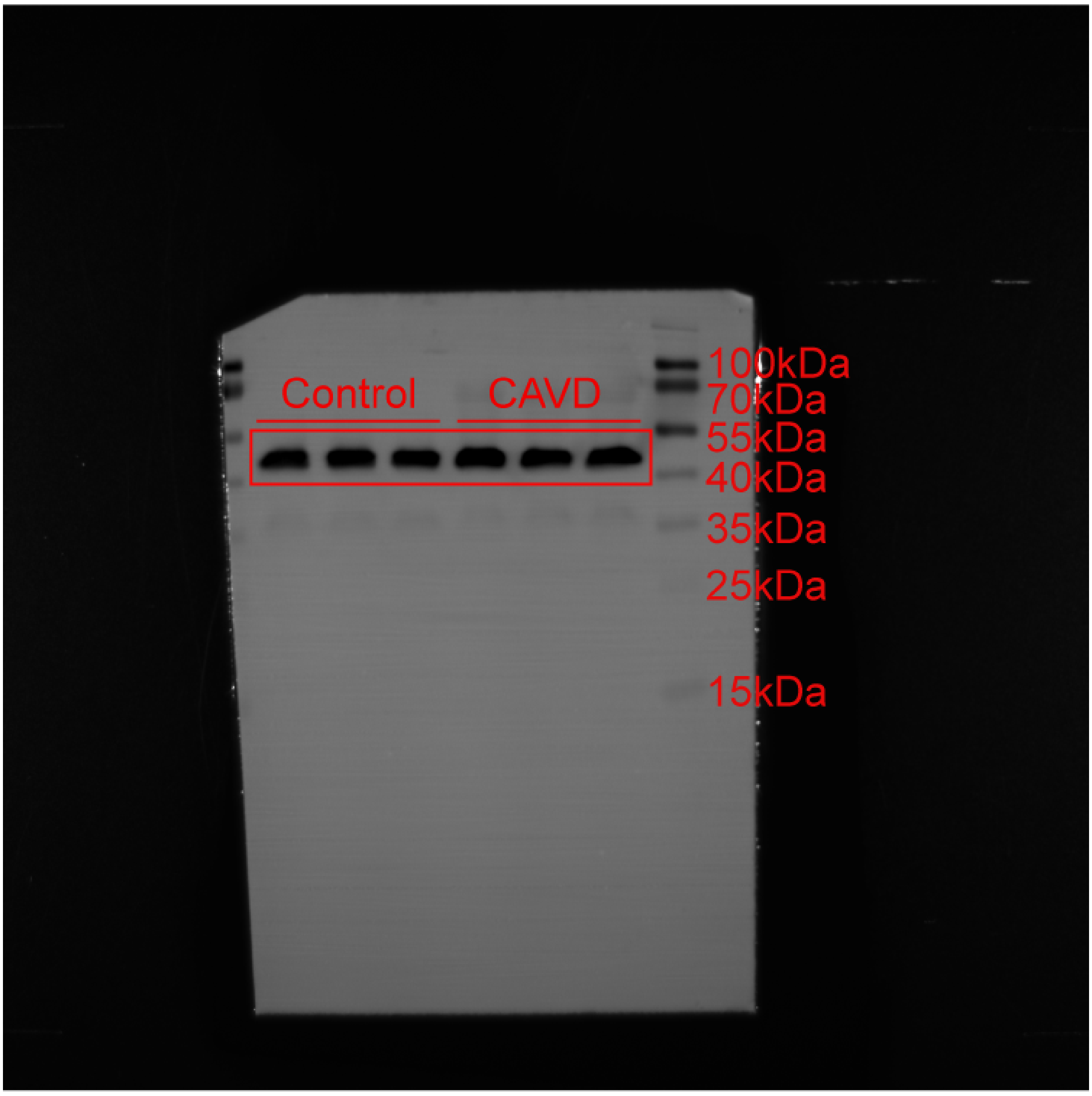

MYOC (55kDa)

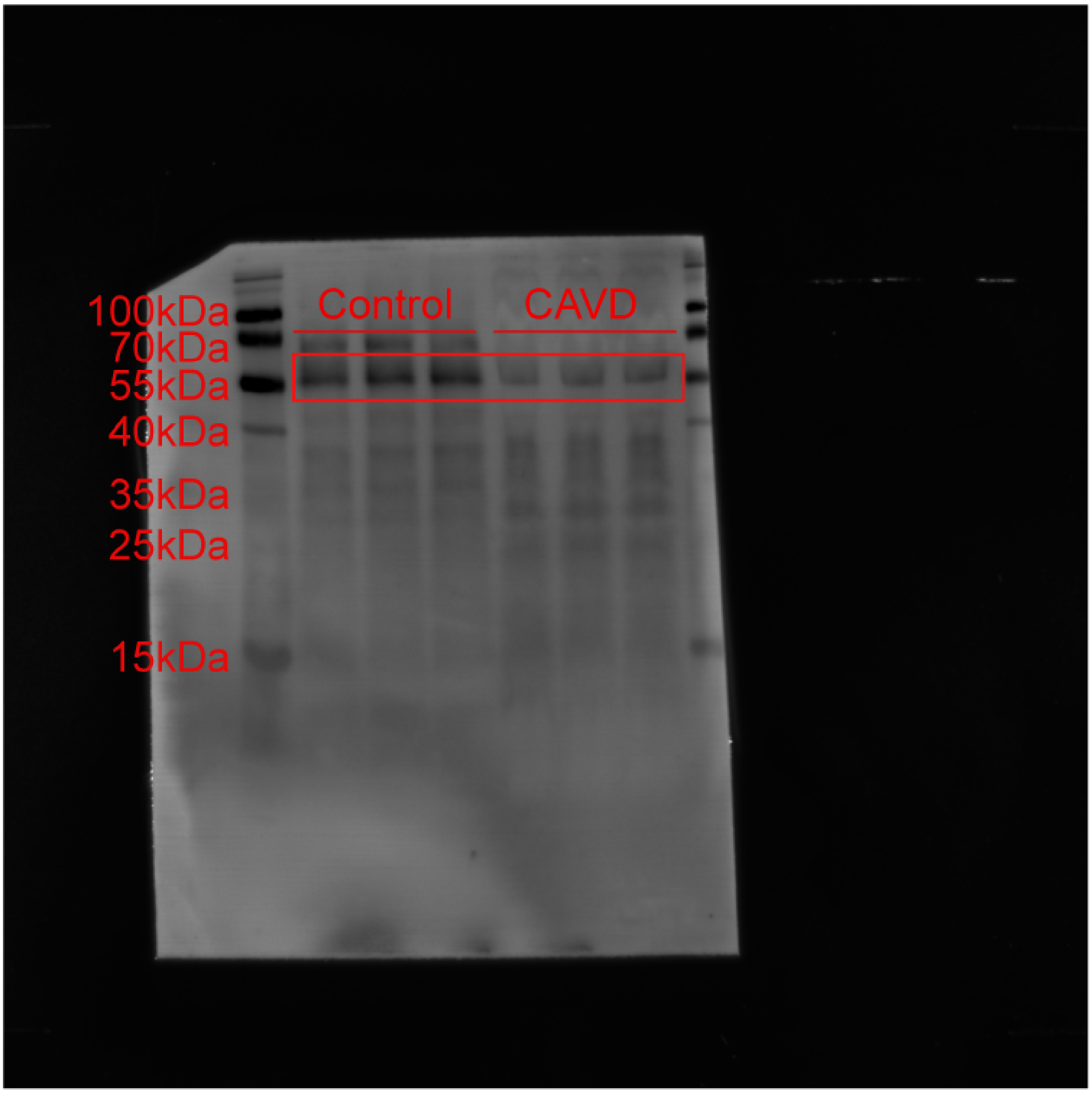

Supplement: Supplementary file 1 [file genes-17-00246-s001.zip › Supplemental meterial S2.pdf]
